# Physician modification of the Terumo Aortic TREO device for juxtarenal or pararenal aortic aneurysms

**DOI:** 10.1016/j.jvscit.2024.101458

**Published:** 2024-02-19

**Authors:** Eric Ducasse, Xavier Berard, Caroline Caradu

**Affiliations:** Unit of Vascular Surgery, Bordeaux University Hospital, Centre Hospitalier Universitaire de Bordeaux, Bordeaux, France

**Keywords:** Endovascular aortic repair, Fenestrated endograft, Physician-modified endograft

We describe the back table modification of a Terumo Aortic TREO device (Terumo Aortic). Combining different cases, our technique of device modification to create manually made fenestrations is presented step by step. This approach enables prompt endovascular interventions for patients with juxta- or pararenal abdominal aortic aneurysms. Access to the fenestrations is typically achieved through the groin. The technique can be adapted to accommodate one to four fenestrations to extend the proximal seal zone.

Although Food and Drug Administration–approved custom-made devices are available, they currently require significant manufacturing time and might not be suitable for all patient anatomies. As an alternative, physician-modified endografts (PMEGs) have been proposed.^1^ PMEGs provide an off-the-shelf option for patients in need of timely repair, yielding excellent mid-term results.^1^ However, concerns persist regarding the complexity involved in planning, graft modification, and target vessel cannulation.^2^ Furthermore, limitations in this technique are attributed to the design features of stent grafts, including narrowly spaced stents that sometimes necessitate placement of a fenestration over a stent strut.^3^

The TREO bifurcated graft (Terumo Aortic) stands out as an endograft with distinctive attributes. It comprises a series of self-expanding serpentine nitinol stents sewn onto tightly woven polyester vascular graft fabric. Notably, its suprarenal fixation stent features five suprarenal and infrarenal barbs oriented caudally. During deployment, the suprarenal barbs are shielded by the clasping system until the main body is fully deployed, and the infrarenal barbs remain concealed within the valley of the first covered stent. This design facilitates safe repositioning until the proximal bare stent is released and facilitates resheathing. This combined fixation pattern, along with an enhanced, narrow-overlapping sealing zone, holds promise in preventing dislodgement and caudal migration.

One notable feature of this endograft is its relatively sparse use of nitinol stents in the main body. The TREO model incorporates only three, four, and five nitinol springs for bifurcate lengths of 80 mm, 100 mm, and 120 mm, respectively, with a strut-to-strut stent distance of ∼18 mm. Also, the stent rows are staggered such that the space between two stent rows is also ∼18 mm. This wide amplitude stent design lends itself to the implementation of fenestrations (Fig 1). For comparison purposes, the Zenith alpha bifurcated graft (Cook Medical) also presents with a strut-to-strut stent distance of ∼18 mm; however, the stent rows are separated by ∼6 mm. The Zenith Flex bifurcated graft and the Zenith TX2 Dissection Endovascular Graft with Pro-Form (Cook Medical) present with a strut-to-strut stent distance of ∼5 mm, and the stent rows are separated by ∼10 mm, which could prove difficult to accommodate multiple fenestrations and large target arteries.

**Fig 1:**
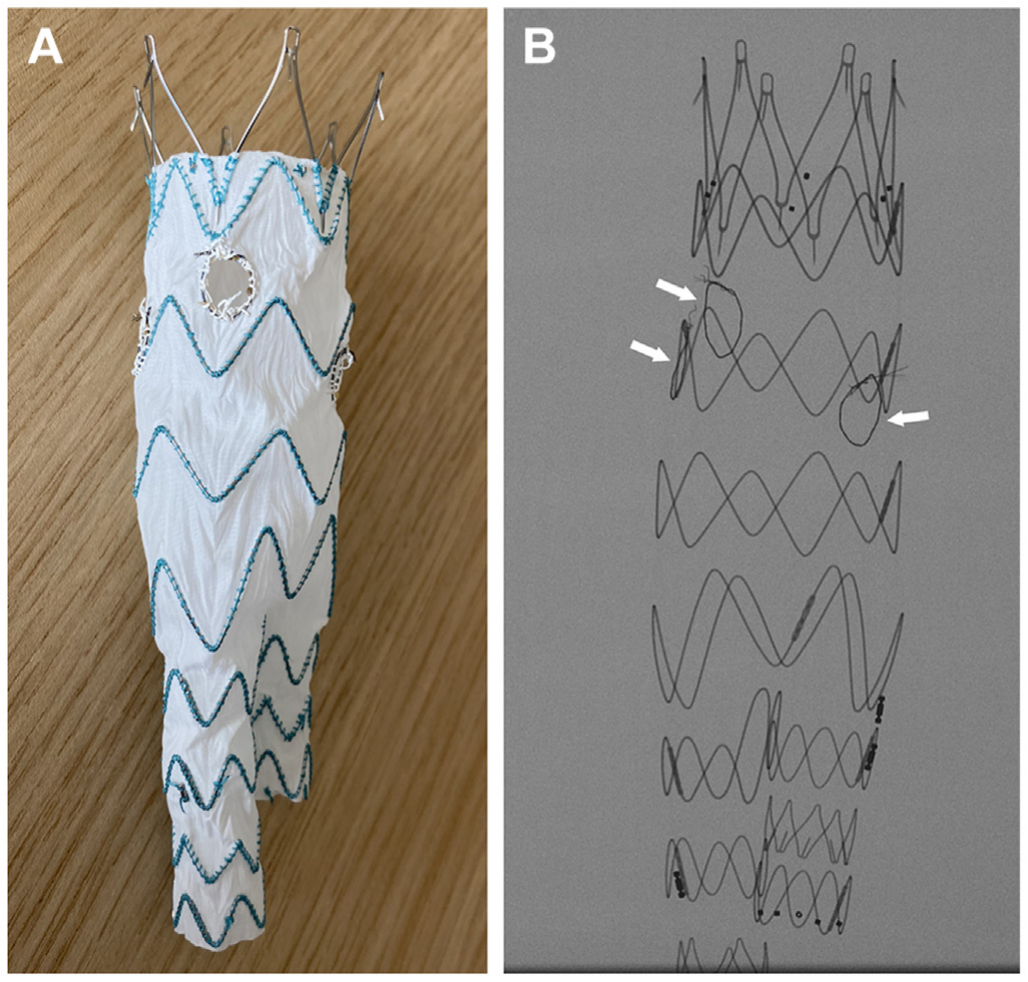
Terumo Aortic TREO device. The wide amplitude stent design and increased interstent distances help physician modification as demonstrated unsheathed on a table (**A**) and radiographically (**B**) in a test device implementing three fenestrations.

The TREO main body has a diameter range of 20 to 30 mm, with increments of 2 mm, and can extend up to a maximum diameter of 36 mm, with increments of 3 mm. The ipsilateral limb is available in three lengths (80 mm, 100 mm, and 120 mm), each with limb gates measuring 14 mm in diameter. The length of overlap between the main body and the limb extensions is highly adjustable, with a minimum overlap of 2 cm on either side and an adjustable zone of 3 cm and 1 cm for the ipsilateral and contralateral limbs, respectively. Another distinctive feature is the unique “lock-stent” mechanism comprising five rounded bars facing intraluminally and positioned at the caudal end of the second most distal stent of the endograft's limb gates. This mechanism ensures modular fixation and mitigates displacement forces, thereby reducing the risk of dissociation and type III endoleaks.

Finally, it is a low-profile device with a delivery system of 18F outer diameter for a 20- to 28-mm main body and 19F outer diameter for a 30- to 36-mm main body. The length from the edge of the covered graft to the delivery handle is 49 cm (with a 67-cm handle), making it possible to accommodate type IV thoracoabdominal aneurysms. In comparison, the Zenith Alpha and Zenith flex bifurcated grafts only offer a 40-cm and 43-cm length, respectively, and the Zenith TX2 Dissection Endovascular Graft, which is a thoracic endograft, offers a 72-cm length (all with a 11-cm handle).

We present a video (Supplementary Video) explaining the different steps to achieve a back table modification of a TREO device (Terumo Aortic) to treat juxta- or pararenal aortic aneurysms. Usually, one member of the team is preparing both sides of the groin with a cutdown on one side to ease the common femoral artery's repair after sheath removal, secondary to our personal experience with PMEGs. With PMEGs, we have tended to observe a slight deterioration of the launcher profile after reinsertion of the PMEG. Percutaneous access is performed on the other side of the groin with preclosure using two ProGlide devices (Abbott Cardiovascular).

During patient preparation, a second member of the team is preparing the PMEG in the operating room according to the preoperative plan. The proximal three to four stents are deployed on the back table. This way, the contralateral gate remains in the sheath.

The fenestration location is identified on the graft fabric, and a sterile pen is used to mark the distance from the proximal fabric edge and the clock position predetermined during computed tomography angiography planning. The diameters of the fenestrations range from 6 to 8 mm in diameter.

Next, an ophthalmic cautery pen is used to create the fenestration in the graft fabric. Subsequently, the marked area is reinforced using an Amplatz goose neck snare (Medtronic) or the proximal tip of a V18 guidewire (Boston Scientific) sutured around the fenestration in a 360° fashion with a CV5 or CV6 Gore-Tex lock-stitch running suture (W.L. Gore & Associates). It is advisable to weigh the benefits of precision and radiopacity and flexibility against the cost and availability; however, we believe our imaging method (Cios Alpha; Siemens Healthcare) has shown that the radiopacity is better with the Amplatz goose neck snare.

The modified endograft is subsequently re-constrained using a silastic lace, which is gradually unwrapped as the delivery system sheath is advanced over the endograft. We acknowledge that it can have an impact on the sheath profile. In our experience, independent of the modified device, this technique can enlarge the sheath, compromising its original manufacturing profile and leading to a small step between the nose and the sheath, which has proved problematic in some percutaneous cases. Some teams have proposed an alternative approach in which the delivery of the PMEG is facilitated through a previously placed DrySeal sheath (W.L. Gore & Associates). However, this method results in an increase of ≥2F in the arterial puncture site. Radiography is conducted before implantation to verify the visibility and orientation of the markers (Fig 2). Device modification requires 20 to 40 minutes, depending on the number of fenestrations.

**Fig 2:**
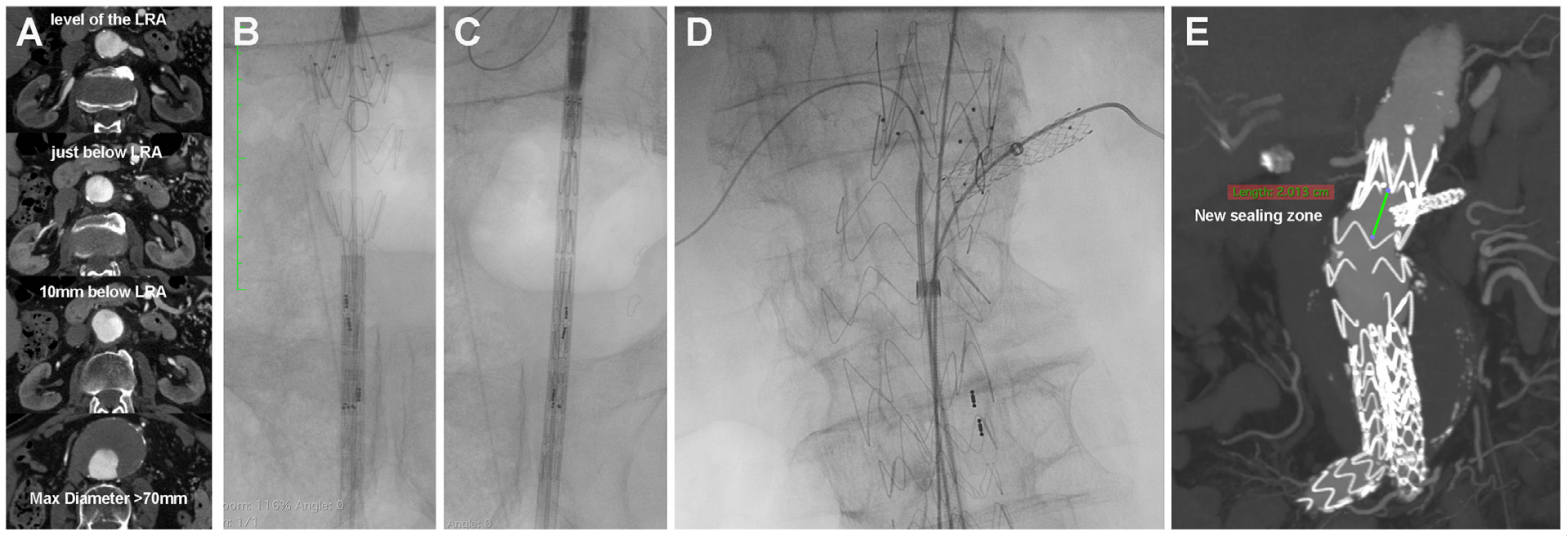
First single fenestration physician-modified TREO device implanted in our center showing the native preoperative computed tomography angiography images at the level of the left renal artery (*LRA*) and below, down to the maximum aortic diameter (**A**), confirming the lack of a proper infrarenal neck. The graft is partially unsheathed after modification under radiographic guidance (**B**) and then resheathed (**C**) and implanted (**D**) to provide a new sealing zone of 20 mm with implementation of a LRA fenestration (**E**).

After endograft deployment, the contralateral gate is cannulated, and a 16F to 20F sheath (Cook Medical) or DrySeal sheath (W.L. Gore & Associates) is advanced inside the main body. The target arteries are routinely cannulated through this large sheath. A 6F to 7F sheath (KCFW; Cook Medical) is advanced, and the target arteries are stented using a balloon expandable iCover stent (iVascular) flared to 9 to 12 mm into the main body.

One technical point is the possibility of graft collapse in the case of rotation of the modified device even after cannulation of the contralateral gate with a large sheath and cannulation of one or more target arteries. On the one hand, it means that no diameter reducing tie is necessary because simply twisting the delivery system handle will allow the graft to torque down to a small diameter, allowing repositioning of the endograft and creation of a gap between the graft and aortic wall if the fenestration is not completely in front of the target vessel's ostium.^4^ On the other hand, it can lead to difficult vessel cannulation because of the presence of graft wrapping inside the main body. Counterclockwise rotation of the graft, along with gentle pulling on the delivery system handle, will bring the graft back to its original shape and help expedite cannulation.

The main body is gradually deployed using a turning knob in the delivery system. This system includes a detachable sheath that can be separated from the main handle, allowing for the introduction of the ipsilateral limb extension. However, we believe this sheath is impractical because of the excessive length and the absence of lateral tubing for injections.

The patient provided written informed consent for the report of their anonymized intraoperative images and videos for educational purposes.

In conclusion, the TREO device's wider amplitude stents and larger interstent distances facilitate modifications, reducing the risk of struts crossing fenestrations. This adjustment does not compromise the technical success rates or safety, because so far the TREO-modified devices (Terumo Aortic) demonstrate comparable success rates, endoleak rates, and major adverse event rates compared with other stent graft types.^4^

## Disclosures

None.

## Supplementary Data

Supplementary Video Physician modification of the Terumo Aortic TREO device for juxta- or pararenal aortic aneurysms.
